# Use of comparative data for integrated cancer services

**DOI:** 10.1186/1472-6963-7-204

**Published:** 2007-12-17

**Authors:** Dawn L Wilkinson, Mark McCarthy

**Affiliations:** 1 Department of Epidemiology and Public Health, University College London, UK

## Abstract

**Background:**

Comparative data are an important resource for management of integrated care. In 2001, the English Department of Health created 34 cancer networks, broadly serving populations of half to three million people, to coordinate cancer services across providers. We have investigated how national and regional routine data are used by the cancer network management teams.

**Methods:**

Telephone interviews using a standardised semi-structured questionnaire were conducted with 68 participants in 29 cancer network teams. Replies were analysed both quantitatively and qualitatively.

**Results:**

While most network teams had a formal information strategy, data were used *ad hoc *more than regularly, and were not thought to be as influential in network decision making as other sources of information. Data collection was more prominent in information strategies than data use. Perceptions of data usefulness were mixed and there were worries over data quality, relevance, and potential misuse. Participants were receptive to the idea of a new limited dataset collating comparative data from currently available routine data sources. Few network structural factors were associated with data use, perceptions of current data, or receptivity to a new dataset.

**Conclusion:**

Comparative data are underused for managing integrated cancer services in England. Managers would welcome more comparative data, but also desired data to be relevant, quality assured and contextualised, and for the teams to be better resourced for data use.

## Background

In England, a Cancer Plan [1] was published in 2000, setting out organizational developments for cancer care in the National Health Service, and creating a new system of 34 cancer networks to serve populations of between half and three million people, so as to integrate services between hospitals and community care. The networks are each managed by a team [2] including a Lead Manager, Lead Clinician, Lead Nurse, and Service Improvement Lead, along with additional Leads and managers (such as Pharmaceutical, Research, and Information) as well as administrative staff.

A national Cancer Information Strategy was also produced to accompany the Cancer Plan [3]. Among recommendations were that 'monitoring of performance indicators which relate to the quality of cancer services delivery, including screening, should form part of the assessment of individual cancer services.' Currently available national and regional comparative data on cancer services may be a potentially important source of information for network teams to fulfil their role. By comparing against their own network's previous performance, the performance of other similar networks, and/or national averages and targets, such data may help the team to guide key decisions taken by senior management within the National Health Service, and internal groups (e.g. the network Board and Executive group).

While administrative data have been used in quality assurance [4] and to compare hospital performance, [5] there has been little research on the availability and use of comparative information for management of integrated services. Previous studies have suggested that 'hard' data may not be the most useful or frequently used source of information in health services, that data may be inadequate and lacking in enough contextual detail to enable comparison [6] and that other sources of information including expertise and personal knowledge might be more influential in decision making [7,8]. A previous qualitative study conducted by the authors, examining four Cancer Networks in England, found that collection of data was of a higher priority than use of data, and locally collected data was of more use in management decision making than nationally aggregated data. In addition, team members perceived barriers to using data, including limited accessibility, limited resources and uncertain data quality. Organizational factors such as roles and remits, relationships within and without the network, and management commitment to using information as a development tool, also impacted upon the use of 'hard' data [9,10].

In the current study we sought to find out how useful currently available national and regional comparative data are for management of the integrated services across all the cancer network teams in England. We investigated how the data are used and the factors that may affect data use. In addition, we wanted to assess possible reactions to providing networks with a dataset collating network relevant cancer information from a variety of currently available routine data sources.

## Methods

Cancer networks coordinate hospital and primary care services that are organizationally linked by their geographical proximity to each other, and cover populations approximating to other health administration and political boundaries in England (e.g. Strategic Health Authority, Cancer Registry, and local government boundaries). The study used telephone interviews of key informants from the management teams of the cancer networks.

### Sample

Following research ethics approval, we sought and received approval from 30 of the 34 network Board Chairs. Twenty nine network teams finally participated in the survey, resulting in 68 individual responses. Reasons were not given by the five network teams for non-participation. Lead Managers, Lead Clinicians and Information/Data Leads were initially approached to be surveyed. In six networks, we were advised (usually by the Lead Manager) to survey 'Other' network team roles including two Service Improvement Leads, a development Manager, a Sector Performance Manager, and a Macmillan Information Lead (for palliative care services). These 'other' roles were usually responsible for network analysis of regional and national comparative data in addition to, or absence of, a Network Information and Data Lead; responses from 'Others' were therefore combined with Information/Data Lead role in the analyses. Seven networks had no Information/Data Lead and no other designated role for looking at data. Two networks had a combined Lead Clinician/Lead Manager (whom we classified as Lead Managers). Full interviews were held with 26 Lead Managers, 18 Lead Clinicians and 24 Information/Data Leads/Others. Three people were interviewed from 14 network teams, two people were interviewed in 11 network teams, and one person was interviewed in four teams.

Participating networks included rural as well as urban settings, and between three and twelve hospital groups and associated community care services. All had a network board including chairman (usually a hospital or community service chief executive), a core network team (including Lead Manager, Lead Clinician, Lead Nurse and Service Improvement Lead), executive representatives from each primary, secondary and tertiary care organisation in the network, a representative from the local health authority, a service user (patient or carer) representative, and a member of the voluntary sector. In addition, some network boards included Lead Cancer Clinicians from primary and secondary/tertiary care providers, a public health representative, a human resource representative, medical school/university representative, financial advisors, and a member of the regional Cancer Registry.

### Procedure

A structured questionnaire was devised based on previous interviews conducted with four network teams [9,10], a previous study of cancer networks in London  [11], and literature on the use of data in health care [7,12]. Advice was gained also from experts in the fields of organisational sociology, health psychology, epidemiology, and statistics, and patient/carer user-representatives. An explanatory letter, study summary and copy of the questionnaire were sent to participants in advance of the telephone interview. Interviews were conducted separately by two research staff and lasted, on average, 30 minutes (ranging between 6 and 45 minutes).

The questionnaire asked about the use of seven data sets available in England, including Cancer Waiting Times, Cancer Registry, Hospital Episodes Statistics, Cancer Standards Peer Review, National Cancer Patient Survey, Healthcare Commission Acute Hospital Portfolio, and Minimum Dataset for Palliative Care [13]. In addition, participants were encouraged to consider any other national and regional comparative data that they used or knew of.

Response options for most of the questionnaire items were yes/no or five or seven point Likert (e.g. ranging from strongly disagree to strongly agree) and semantic differential response scales (e.g. ranging from useless to useful). Participants were able and encouraged to provide additional feedback and comments throughout the survey (full questionnaire available on request from the first author). We also recorded some information on network structures and contexts directly from the network teams and from a national cancer data resource [14].

Qualitative responses to open ended questions were transcribed, ordered by question number, and examined to identify common themes. Once categorized, responses were content analysed (counting the same or similar responses) to indicate frequency of beliefs/examples across participants (i.e. in relation to: sources of data, uses of data, reasons for sharing data with the Network Board, and barriers to using data). Qualitative responses also enabled the identification of answers that had not been pre-empted in the structured questionnaire (e.g. reasons for not providing user-groups with data), and provided elaboration and anecdotal examples to complement the quantitative data (e.g. reasons for high/low receptivity to a new comparative data set). Examples and issues arising from qualitative data have been summarised in the results and where appropriate, we present the statistical data supported by participants' comments.

To summarise findings, five point and seven point response scales were divided into negative, neutral, and positive responses. Frequencies and percentages refer to individual level analysis unless stated otherwise. Data were analysed individually, by role, and aggregated (mean average response across participants) by network. Differences between roles are taken to imply general level of concordance within teams, and substantial differences in opinions within teams are highlighted. Some correlational analyses (Pearson's r for parametric data and Spearman's Rho for non parametric data) were conducted to identify possible relationships between data use, perceptions of data, and organisational factors. However, as there were only 29 networks and 68 individual participants, statistical power to detect an effect is weak, and general trends are mainly reported.

## Results

### Data use across the network teams

• Extent of data recognition and use

Two data sets, Cancer Waiting Times and Cancer Registry, were the data sources most often recognised and most often reported as being used by the network teams (Table 1). This was the case across all three roles.

**Table 1:** Study participants' awareness and use of nationally available data sources.

		**Aware of**		**Use**	
**Data source**	**Characteristics**	**No. participants**	**%**	**No. participants**	**%**

Cancer Waiting Times Database	Times for patients referred by general practitioners as 'urgent' (216 hospitals)	68	100	66	97
Cancer Registry	National linkage of cancer registration with death certificates	68	100	63	92
Hospital Episodes Statistics	Routine data on NHS patients – 12 million episodes per year	67	98	45	66
Cancer Standards Peer Review	One-off survey to assess standards in 110 cancer 'units' and 46 'centres'	64	94	40	58
National Cancer Patient Survey	Sample survey of patients discharged from 172 acute hospital services	56	82	40	58
Minimum Dataset for Palliative Care	Staffing and activity of palliative services	49	72	19	27
Healthcare Commission Acute Hospital Portfolio	Staffing and facilities in 188 NHS hospital trusts	41	60	8	12

In addition to the seven specified data sources, 60% (41/68) participants reported using a total of 24 other (identifiable) types of data that enable comparison (with national averages, and/or across hospital trusts and/or networks); 20 of these could be allocated to a single national source (rather than generic data such as clinical research/evidence-based data) and are specified in Table 2. Participants also reported using locally collected data and local health administration data.

**Table 2:** Additional nationally available information sources used by the participants

**Information**	**Mentions**	**Source**	**Description**
National Clinical Audit Support Programme	8	Healthcare Commission	Tumour-specific local audits
Secondary analysis of cancer registry data	7	Office of National Statistics	National publications of cancer registrations and survival.
NatCanSat data	7	National Cancer Services Analysis Team	Resource for data on services provision and activity.
Cancer screening data	7	NHS Cancer Screening Programmes	Breast, cervical, prostate and colon cancer screening.
National reports on cancer services	6	National Audit Office	Parliamentary reports, with some secondary analyses
Professional Body/Royal Colleges	5	Royal College of Surgeons and specialty groups	Professional bodies carry out audits.
Smoking cessation data	4	NHS	Stop smoking services statistical bulleting published annually.
Cancer drugs approvals	4	National Institute for Health and Clinical Excellence	Cancer drugs in association with NHS National Cancer Director
Web-based Information Learning System (WILS)	4	NHS Cancer Services Collaborative	Organisational and tumour specific information for managers
Dr Foster	4	Commerical provider	Analysing and presentation of NHS data.
Star ratings data	2	Healthcare Commission	Performance indicators for monitoring health care.

Others with one mention: National Centre for Health Outcomes Development; National confidential enquiry into patient outcome and death; Improving Outcomes Guidance data; Gold Standards Framework; Public Health Observatory; Pharmaceuticals utilization data; Pathspeak – pathology data; Programme budgeting data; National tracking exercise for investment in cancer services.

Total use of the seven data sources was positively (although not significantly) correlated to use of self-reported additional data sources (Pearson's r = 0.31 p > 0.10) suggesting that low use of the seven data sources was not a result of dependence on other sources of data, and that those using fewer of the seven data sources, used less data generally. At the network team level (mean use across network respondents) average use was rated, on a 7 point scale, at just over 4 (s.d. = 0.90, range 2.5 to 7) data sources. Average use was higher amongst Lead Clinicians (mean = 4.4) than either Lead Managers (mean = 4.2) or Information/Data Leads and Others (mean = 3.7).

• Strategies and routines

Over half the participants (59% 40/68) indicated that their network team had some sort of formal information strategy, although only 34% (23/68) thought it covered issues of local data collection *and *data use equally. Only one participant thought their strategy mainly covered data use, whilst 23% (16/68) indicated that the main focus was on data collection. There was some disagreement between respondents within teams (in 9 out of 25 (36%) network teams that had more than one participant), over whether an information strategy existed and over the focus of the strategy. A higher percentage (72%, 13/18) of Lead Clinicians thought that the team had an information strategy than either Lead Managers (54%, 14/26) or Information/ Data Leads and Others (54%, 13/24). Despite having information strategies only 26% (18/68) participants said that they were most likely to use data routinely (as opposed to ad hoc). However, frequency of data use depended largely on the data source in question with Cancer Waiting Times data being looked at weekly, daily and "*almost hourly*".

• Specific uses

Data were used by network teams for three required activities [15] – implementing guidance on clinical practice in cancer services (76%, 52/68 participants indicated use), service improvement initiatives (66%, 45/68 indicated use) and to undertake a national peer review of cancer service standards (44%, 30/68 indicated use). Cancer Registry data and Hospital Episodes Statistics were most frequently used for implementing Improving Outcomes Guidance whilst Cancer Waiting Times and Registry data were most frequently used for service improvement plans.

Participants were also asked to think of other examples of data use. The most commonly cited general uses included planning (8 participants), providing supporting evidence (8 participants), and setting the network picture in a national context (6 participants). Frequently cited specific uses included validating and checking other data (e.g. Cancer Registry and Cancer Waiting Times) (9 participants), assessing treatment trends (e.g. Hospital Episodes Statistics) (8 participants), and monitoring waiting times in order to meet targets (8 participants).

• Dissemination

Dissemination of data to key network stakeholders, including Board members and cancer patient and carer/user representatives, was occurring although not consistently within or across teams. Fifty three percent (36/68) of participants reported that their networks (but not necessarily network team) had provided some national and regional comparative data to patient and carer user groups in the past year with Cancer Waiting Times and Cancer Registry data being the data sources cited most frequently. Data were unlikely to be disseminated routinely but more likely in relation to specific initiatives, nationally released documents, or as and when the users asked for data. The most frequently cited reasons for not providing data to user groups were that there were currently no means or forums to feed data back, or that the user groups were still developing and deciding their own priorities (see Table 3). Reasons were similar across roles although Lead Managers were more likely than other roles to say that they had not thought about it or got around to it, whilst Lead Clinicians, Information/Data Leads and Others, more often said that users had not asked for the data and/or would not want them anyway.

**Table 3:** Main reasons for data not having been disseminated to user groups

**Reason**	**No. of participants reporting reason**^§^
No means or forum to present the data to user groups	8
User groups are not ready	10
Data are not relevant to user groups	3
Users would not understand the data	3
Users haven't asked for the data/wouldn't want it	4
We just haven't thought about it/got around to it	4
Other	2
Don't Know	2
N/A	46

^§^Reasons were given by the participants and were not fixed response options. Participants could give more than one answer.

Fifty four percent of participants (37/68) noted that national and regional comparative data were discussed at Board meetings. Data were discussed to: keep board members informed; contextualise local/network data; or be set aside (as problematic). National and regional comparative data were seen to be less influential on average in guiding strategic decisions taken by the network/network Board, than other factors and sources of information including: relationships between decision makers; knowledge, experience and expertise; and locally collected data.

#### What factors may affect the use of national and regional comparative data?

• Perceived usefulness

We asked the participants to rank data source usefulness and found that Waiting Times and Cancer Registry data were ranked as most useful by the majority of participants. This apparently related to current network priorities and national targets:

"the Cancer Waiting Times is a big focus of our work at the moment but that reflects political priorities"

In addition, five participants noted that locally collected data were often more useful than national and regional comparative data due to their specificity and relevance.

Participants were asked to indicate whether national and regional comparative data enabled the network team to assess network quality of service. Results indicated a mixed response with 50% (34/68) indicating that the data were useful in this way. Fewer Lead Clinicians thought that the data assessed quality of service compared to Lead Managers or Information/Data Leads and Others (22% (4/18) vs 65% (17/26) and 72% (13/18) respectively). At network team level average perception (out of 5) was just over 3 (neither agree/nor disagree that currently available data enables the network team to assess network quality of service), with 15 networks scoring the midpoint or lower. Many participants questioned the extent to which currently available data enabled them to monitor the service at the level of multi-disciplinary team (groups of health professionals at the hospital trust level who are ultimately responsible for delivering services). Only 35% (24/68) said that the data were useful at this level. The most positive attitudes were among Lead Managers (46% (12/26), saying the data were useful, compared to only 28% (5/18) of Lead Clinicians and 39% (7/18) Information/Data Leads/Others respectively.

• Perceived barriers

More than half the respondents (57%, 39/68) thought that people in their network do not generally trust the quality of available data. This figure was higher amongst Lead Clinicians (66.7% 12/18). However, they considered that trust was increasing. Two thirds (65%, 44/68) thought that people in their network worry about how comparative data could be used, reporting worries of simplification, misinterpretation, and suspicion that data could be used against them (e.g. to withdraw resources). Again, this was most common amongst Lead Clinicians (83% 15/18). Sixty percent (41/68) of participants thought that the relevance of some data is not always clear; however, one participant acknowledged that the data were relevant but said that "*we are not that clever at using some data*". More Lead Clinicians thought that the data were not relevant (78% 14/18) than Lead Managers (58% 15/26) or Information/Data Leads/Others (67% 12/18).

Rather fewer respondents thought that Government priorities prevented them from using data as they would like to, with just over 35% (24/68) responding "neither/nor" and the remainder divided fairly equally between agreeing and disagreeing. In response to an open question around barriers to data use a broader range of issues were also proposed as preventing respondents from using data as they would like to, including poor or uncertain data quality (16 participants), lack of timeliness and/or being out of date (13 participants), general distrust of the data (8 participants), and not being able to access data sources easily/not receiving data routinely or automatically (16 participants).

• Receptivity to new comparative data

We were particularly interested in what participants would think about being given a new set of aggregated cancer data to allow comparisons of performance across hospitals within their network and nationally. The large majority (87%, 59/68) thought such a dataset would be a good rather than bad idea: 81% (55/68) thought it could be useful for monitoring quality of service at network level, 78% (53/68) thought it could be useful for monitoring quality of service at hospital trust level, and 88% (60/68) thought that it could be useful in their role. However, only half (49%, 33/68) of participants thought that such as dataset would be welcomed by others, and only 21% (14/68) thought that the data would be trusted by clinicians. Creating a composite measure of 'receptivity to limited dataset' (mean score of all six items on perceptions of a new comparative limited dataset), the average score across network teams was 5.2 out of 7 (with 7 indicating higher receptivity), but team averages ranged from lowest 2.8 to highest 6.5.

Concerns about the dataset largely related to inadequate resources: participants expressed concerns about what such a dataset would mean in the long term and how its collection would be overseen. They also wanted to know how labour intensive it would be to analyse it:

"the amount of time it would take to produce, the amount of time spent collecting and justifying it and the amount of arguments over it would not be worth it...it would make my life [as Information/Data Lead] more difficult"

Concerns were also raised about potential misinterpretation and misuse:

*"it could be incredibly damaging [if not contextualised]*"

Thus participants made a number of recommendations if such a dataset were to be accepted and used effectively by network teams. These included: the importance of considering contextual detail; the importance of assessing, and giving assurance about, the quality of the data; and presenting the data as a prompt for further investigation rather than as a definitive judgment of quality.

#### Relationships between data use, perceptions, and network context

We examined whether perceptions of usefulness in assessing quality, barriers to using the data, and receptivity to a new limited dataset, were associated with the use of seven key data sources by the network team. In addition, we also examined whether data use and perceptions of data were associated with individual and organisational network factors including length of time respondent has been in their role, presence of an information lead in the network, cancer population of network area, number of Strategic Health Authorities (performance management bodies) in network, number of Primary Care Trusts covered by the network, number of Hospital Trusts covered by the network, and whether the network crosses Cancer Registry regional boundaries (see Table 4). We created four composite measures of perceptions of data sets: 'Total use' (additive score of the number of data sources used, out of 7); 'Assess quality' (mean score of answers to 'Perceptions of the usefulness of current data to assess quality of network service'); 'Perceived barriers' (mean score of answers to "People in this network do not trust the quality of the data" and "People in this cancer network worry about how data will be used"); and 'Receptivity to limited dataset' (mean score of all six items on perceptions of a new comparative limited dataset). Pearson's r correlations were used for normally distributed interval level data and Spearmans Rho correlations were used for non non-normally distributed and ordinal level data. Associations were examined at individual and network level. Relationship trends and significant associations were the same at network and individual level (only network level will be reported). Small sample sizes led to few significant findings.

**Table 4:** Network level correlations: data use, perceptions about data use, and organisational contextual factors

	1	2	3	4
1. Total use	1			
2. Assess quality	-.04	1		
3. Perceived barriers	.13	-.17	1	
4. Limited dataset receptivity	-.46*	.52**	-.37*	1
5. Time in role	.24	-.22	.43*	-.41*
6. Cancer population	-.24	-.04	.21	.13
7. No. of SHAs	.06	.07	.08	-.01
8. No. of PCTs	.11	.10	.13	.13
9. No. of hospital trusts	.14	.29	-.06	.24
10. Crosses Registry region	.28	.30	-.06	-.07
11. Presence of Info-Lead	-.06	.13	.11	-.06

*p < .05 **p < .01 ***p < .001

The composite value 'Receptivity to limited dataset' was negatively correlated with 'Total use' (rho = -0.46, p < 0.05), suggesting that the more data sources currently used by the team, the less receptive network teams are to a new dataset. It was also positively associated with 'Assess quality' (rho = 0.52 p < 0.01) and negatively associated with 'Perceived barriers' (rho = -0.37 p < 0.05) suggesting that respondents who perceive that currently available comparative data allow them to assess service quality, and perceive few barriers to using the data, are more receptive to a new dataset.

Small (although non-significant) effects were also seen for some organisational contextual factors including data use with: average length time in post (r = .24), cancer population size of the network (-.24) and the network crosses a Registry region (r = .28). No other organisational factors appeared to be related to data use. Time in post was also positively correlated with perceived barriers to using data (rho = .43 p < .05) and negatively correlated with receptivity to limited dataset (rho = -.41 p < .05) (and this was particularly so amongst Lead Clinicians).

## Discussion

Cancer networks are an important innovation in integrated management of health services in England. However, the information strategy for cancer [3] addressed primarily clinical and administrative data within hospitals and at national level, rather than promotion of integrated care through the cancer networks. We identified information strategies in only just over half the networks. Participants in our study were concerned about data quality, and relevance to their particular areas of work. They were, however, generally receptive to new comparative data.

Use of the seven national data sets relating to cancer services that we described varied from over 90% for Cancer Waiting Times and Cancer Registry data, to less than 30% the Healthcare Commission Acute Hospital Portfolio and Minimum Data Set. Use seemed to be closely related to current national cancer services targets and directives, with networks prioritizing data relating to immediate performance issues on which networks could be held accountable. Similar 'tunnel vision' effects have been observed in other studies of information use, for example in performance indicators [16] and the Government's focus on waiting time targets has been found in other services to have complex, perhaps unexpected, clinical impacts [17].

Local data, individual expertise, and organisational and individual relationships were more important than comparative data in decision making and have been shown elsewhere to be a major influence in healthcare management [7,8]. Only half the respondents thought that currently available national or regional comparative data enabled them to assess the quality of service in their network and fewer than 40% of respondents always saw the relevance of currently available data. There needs to be more discussion between data collation agencies and those expected to use the data (e.g. managers, decision makers, policy makers) to ensure that data being collected include measures that are valuable and useful.

Our respondents were concerned about the quality of national data sets, and how data may be used. This lack of trust appeared in the interviews to be most acute amongst network clinicians. Lead Clinicians more often reported that data sets were of little use and expressed their lack of trust in them. Resistance to data use by practitioners has been well documented [7,18,19]. However, greater use of data sets can contribute to recognition of their place in NHS management, while more discussion is needed with clinicians in supporting their own information needs. Statistical analysis of the responses from team members is, however, limited by the size of the samples.

Members in longer serving roles reported more use of data but also had stronger perceived barriers to using data and were less receptive to a new limited dataset. These associations were greater for the Lead Clinicians. Few other organisational or network contextual factors were associated with data use or perceptions, and it is possible to suppose that greater encouragement by government to use datasets for management would be accepted. Cancer networks with smaller cancer populations and those crossing health care administrative boundaries tended to be more positive towards using data. Other contextual factors, for example network budgets, management ethos, and team relationships and structures might be investigated in future as determinants of information use.

## Conclusion

Comparative data are underused for managing integrated cancer services in England. The priority given by cancer network managers to collection of data by services, rather than to the use of data for management, reflected the priorities of central government, which had set targets for completeness (especially for waiting times) rather than supporting networks in the more complex role of understanding and interpreting available data.

Networks need to be encouraged to develop and use information strategies that deal not only with technical issues and processes of collection, but also with approaches for using data and turning it into meaningful information and action. Increased engagement with data within the network teams may lead to increased dissemination and information sharing amongst other cancer network stakeholders. Promisingly, our results showed that network team members would be receptive to new comparative data collating information from currently available data sources, and would find it useful in their roles. There is reason to believe that such data would be used if the teams were better resourced for data management, if the data were relevant, quality assured and contextualised with network-specific information.

## Competing interests

The author(s) declare that they have no competing interests.

## Authors' contributions

DW carried out the design of the questionnaires, interviews, analysis of data, and writing up the paper. MM conceived of the study, participated in its design and coordination and contributed to writing up. Both authors read and approved the final manuscript.

## Pre-publication history

The pre-publication history for this paper can be accessed here:


